# Low intensity extracorporeal shockwave therapy for chronic pelvic pain syndrome patients with erectile dysfunction

**DOI:** 10.1097/MD.0000000000028546

**Published:** 2022-01-14

**Authors:** Datesh Daneshwar, Abid Nordin

**Affiliations:** a Urology Clinic, Prince Court Medical Centre, 39, Jalan Kia Peng, Kuala Lumpur, Malaysia; b MedCentral Consulting, B-1-4, Desa Bangsawan, Jalan 27/117A, Bandar Tun Razak, Cheras, Kuala Lumpur, Malaysia.

**Keywords:** chronic pelvic pain syndrome, erectile dysfunction, low intensity shock wave therapy, quality of life, urinary symptoms

## Abstract

**Introduction:**

In this study, the efficacy of low intensity shock wave therapy (LSWT) in improving symptoms of chronic pelvic pain syndrome (CPPS) and erectile dysfunction (ED) was investigated.

**Methods:**

Men diagnosed with CPPS and ED (n = 50) were prescribed with LSWT. The LSWT was administered in 10 sessions over the course of 5 weeks at 3,000 pulses with .25 mJ/mm^2^ energy flow and 5 Hz frequency. Outcome parameters were measured before and after LSWT.

**Results:**

Clinical symptoms related to CPPS and ED were measured using four validated questionnaires namely National Institute of Health Chronic Prostatitis Symptom Index (NIH-CPSI), the International Index of Erectile Function (IIEF), the International Prostate Symptom Score (IPSS), and Sexual Health Inventory for Men (SHIM). The effect of LSWT on each of the three domains of NIH-CPSI, namely Pain, Symptoms, and Quality of Life (QoL) were also analyzed. Uroflowmetry was measured to assess LSWT effect on urine voiding. The mean baseline CPPS symptoms on NIH-CPSI domains of pain, symptoms and QoL were 9.92 ± 5.72 (mean ± SD), 5.14 ± 14.5, and 8.02 ± 3.17, respectively. LSWT resulted in significant reduction of CPPS symptoms on all NIH-CPSI domains (Pain = .9 ± 1.37; Symptoms = .74 ± 1.03; QoL = 1.16 ± 1.78). The baseline means of CPPS symptoms on IIEF, IPSS, and SHIM were 45.42 ± 16.24, 24.68 ± 9.28, and 14.28 ± 6.02, respectively. LSWT significant improved CPPS symptoms on IIEF (49.48 ± 28.30) and IPSS (9.04 ± 7.01) but not on SHIM (16.02 ± 9.85). No statistically significant differences were observed with all uroflowmetry parameters.

**Conclusion:**

The current study demonstrated for the first time the safety and efficacy of LSWT administered in 10 sessions over 5 weeks in improving symptoms of CPPS and ED without causing any significant adverse effect to the patient.

## 1 Introduction

Chronic pelvic pain syndrome (CPPS) is a common occurrence among adult men, with an estimated prevalence of 2.7% to 16%.^[1]^ Erectile dysfunction (ED) affects most men who are suffering from CPPS. Although the exact underlying mechanisms are unclear, CPPS is thought to be associated with increased risk factors of ED—arterial stiffness and endothelial dysfunction. Besides, psychological factors also contribute to ED in CPPS patients. This is because CPPS patients suffer from considerable stress, depression, and anxiety, which together with the pain symptoms and voiding dysfunction of CPPS lead to decreased sexual activity and erectile function.^[2]^ A recent study by Crocetto et al^[3]^ even suggested the association of CPPS with burning mouth syndrome (BMS), a medical condition characterized by the burning and painful sensation in the oral cavity without any visible wounds or lesions. Like CPPS, the exact etiopathogenesis of BMS is unknown and seems to be complicated with suspected interactions between local, systemic, and psychogenic factors. A theory suggested that BMS may be due to either peripheral nerve damage or dopaminergic system disorders, which suggested a neuropathic characteristic in BMS and thus, probably gives rise to CPPS.^[4]^ Nonetheless, more studies need to be conducted to confirm the co-occurrence of CPPS with BMS since the study by Crocetto et al^[3]^ was the first study to demonstrate a connection between CPPS and BMS.

Despite the ubiquitous nature of CPPS, consensus on a specific treatment for its management is still lacking. Among treatment managements for CPPS include various anti-inflammatory agents, analgesics, antibiotics, α-receptor blockers, and 5α-reductase inhibitors, either to be used separately or in combination.^[5]^ To date, efficacy of each treatment type is still not established with mixed outcome in numerous studies.

A shock wave is a continuous transmission of sound wave, carrying energy that propagates through a medium, and terminates in a burst of energy, similar to a mini-explosion.^[6]^ In medical science, shock wave has been utilized to break aggregated deposits within the tissue such as kidney stones.^[7]^ At low intensity, shock wave was found to induce cell proliferation,^[8]^ angiogenesis,^[9]^ and nerve regeneration.^[10]^ These benefits of the low intensity shock wave were found to be due to the modulation of various mechanisms, depending on tissue type and condition.^[11]^ Consequently, low intensity shock wave therapy (LSWT) has been used to treat musculoskeletal disorders,^[12]^ nonhealing wounds,^[13]^ and myocardial infarction.^[14]^

In urology, LSWT is a well-known treatment for ED, with its popularity continue to grow over time.^[15]^ Additionally, the body of literature on LSWT application for other indications within urology, keep on expanding over the years. Other indications include prostatic hyperplasia,^[16]^ Peyronie's disease,^[17]^ stress-induced urinary incontinence,^[18]^ and overactive bladder.^[19]^ More recently, interest in the utility of LSWT in CPPS is growing. Numerous studies, either randomized or non-randomized, reported a significant improvement of CPPS clinical symptoms following LSWT.^[20–29]^

Like many frontier therapies in the early stage, consensus on the treatment modalities of LSWT in CPPS has not yet been established. Majority of the studies delivered their LSWT in 4 sessions over the course of 4 weeks.^[20–27]^ Zhang et al^[28]^ expanded their sessions into 8 over the week. In terms of the parameters of the shock, majority setting was at 3000 pulses with .25 mJ/mm^2^ energy flow density (EFD) and 3 Hz frequency. Some other settings include 5000 pulses with .096 mJ/mm^2^ EFD and 5 Hz frequency,^[25]^ 2000 pulses with .14 mJ/mm^2^ EFD and 3 Hz frequency,^[29]^ and 2500 pulses with .25 mJ/mm^2^ EFD and 3 Hz frequency.^[21,22]^ In the current study, efficacy of LSWT was investigated among CPPS patient with ED, administered in 10 sessions over the course of 5 weeks at 3000 pulses with .25 mJ/mm^2^ energy flow and 5 Hz frequency, on improving the clinical symptoms of CPPS and ED was evaluated.

## 2 Methods

### 2.1 Study design

A prospective interventional study was conducted between May 2018 and February 2021 on patients who attended urology clinic with chronic pelvic pain syndromes (CPPS) receiving low intensity shockwave therapy (LSWT). All efforts were taken to comply with the ethical principles outlined in the Declaration of Helsinki and Good Clinical Practice Guideline.

### 2.2 Eligibility criteria

Inclusion criteria were all patients attending the urology clinic with symptoms of CPPS (type IIIB), namely complaints of pain or discomfort in the perineal or pelvic region for at least a 3 months period within the last 6 months without clear abnormalities on urological examination and consented for the study. Exclusion criteria were patients who refused LSWT, defaulted follow up post LSWT and patients who had not given any consent.

### 2.3 Data collection

Patients were assessed for symptoms of CPPS with the National Institute of Health Chronic Prostatitis Symptom Index (NIH-CPSI) and the International Prostate Symptom Score (IPSS) questionnaires; for symptoms of ED with the sexual health inventory for men (SHIM) questionnaire; and for qualities of sexual functions (erectile function, orgasmic function, sexual desire and intercourse satisfaction) with the International Index of Erectile Function (IIEF) questionnaire. Additionally, data on uroflowmetry outcome was assessed when available. All the assessments were made pre-intervention and repeated post-intervention. Data was collected and organized in SPSS (v22, SPSS Inc., IBM) for data analysis. Patient's information and data were kept confidential before and after data collection. Patients who showed improvement in either 1 of the above mentioned parameters (NIH-CPSI, IPSS, SHIM, IIEF, and uroflowmetry) were regarded as a successful experimental group whereas patients who did not show any improvement in all of the parameters above were considered as a failed experimental group.

### 2.4 Treatment protocol

Prior to the LSWT treatments, the patients were prescribed with either 500 mg of levofloxacin once daily or 500 mg of ciprofloxacin twice a day for 1/12 along with alpha blockers, Harnal or Tamsolusin. Their CPPS symptoms recurred and they were then treated with 5 mg of Cialis once daily for a month in combination with the LSWT treatments. The LSWT treatments were given twice a week for 10 sessions over the duration of 5 weeks in an outpatient setting without local or systemic anesthesia. At each therapy session, 3000 impulses were applied on the perineum, with a total energy flow density of .25 mJ/mm^2^, 5 Hz (Duolith SD1 Ultra, Storz Medical AG). Follow-up was done 1/12 post LSWT treatment and the patients were not on any medication then.

### 2.5 Statistical analysis

SPSS (v22, SPSS Inc.) was used for all statistical analyses. Descriptive data were expressed as mean ± standard deviation (SD). Kolmogorov-Smirnov test was used to assess the normal distribution of data. The statistical differences between pretreatment and post-treatment was assessed with paired *t* test for normally distributed data and Wilcoxon signed rank test was used to analyze the non-normally distributed data. A value of *P* < .05 is considered statistically significant.

## 3 Results

A total of 50 patients were included in the current study. The mean age of the patients is 41.9 ± 11.7 years old while the distribution of patients according to ethnicity were made up of 22 (44%) Malay, 8 (16%) Chinese, 11 (22%) Indian, and another 9 (18%) noted as other ethnicity. According to the collective responses from the questionnaires (NIH-CPSI, IPSS, IIEF, and SHIM), the presenting signs and symptoms reported by patients include pain/discomfort in the area between rectum and perineum, testicles, tip of the penis and pubic or bladder area; pain/burning sensation during urination and ejaculation; incomplete voiding sensation; frequency, intermittency, urgency, weak stream, and straining during urination as well as ED.

Before the LSWT therapy, the patients were prescribed with antibiotics such as levofloxacin or ciprofloxacin in combination with alpha blockers such as Harnal or Tamsolusin to relieve the symptoms of CPPS. However, the symptoms recurred and they were then treated with Cialis coupled with the LSWT therapy. After completion of the 10 weeks session of LSWT, all measured outcomes were significantly improved, except the SHIM outcome as depicted in Table 1. In brief, LSWT reduces the score of NIH-CPSI in the pain (*P* < .001), symptoms (*P* < .001), and quality of life domains (*P* < .001). Next, 10-sessions of LSWT reduces the total score on IPSS questionnaire (*P* < .001). For IIEF questionnaire, LSWT significantly increases the total score after 10-sessions (*P* < .001). Uroflowmetry outcome were available for 47 patients before LSWT and 26 patients after LSWT. The peak flow rate, void volume, and voiding time were not affected by LSWT treatment (Table 2). No significant side effects were observed during the whole course of treatment. In general, all 50 patients showed significant improvements in NIH-CPSI, IPSS, and IIEF but not in SHIM after LSWT treatment. As for the uroflowmetry outcome, the 26 patients whose data were available reported no significant changes in the parameter after LSWT treatment. Despite some outcomes like SHIM and uroflowmetry which showed no significant changes after LSWT treatment, the significant improvements shown by other outcomes like NIH-CPSI, IPSS and IIEF indicated an overall success of the treatment in improving the symptoms of CPPS, including pain/discomfort in the genitalia, urinary symptoms, ED, and quality of life in all the 50 patients.

**Table 1:** Summary of changes in clinical symptoms following low intensity shock wave therapy.

	NIH-CPSI					
Instrument	Pain	Symptoms	QoL	IPSS	IIEF	SHIM
Pre-LSWT	9.92 ± 5.72	5.14 ± 14.5	8.02 ± 3.17	24.68 ± 9.28	45.42 ± 16.24	14.28 ± 6.02
Post-LSWT	.9 ± 1.37	.74 ± 1.03	1.16 ± 1.78	9.04 ± 7.01	49.48 ± 28.30	16.02 ± 9.85
*P* value	<.001^∗^	<.001^∗^	<.001^∗^	<.001^∗^	.036^∗^	.130

IIEF = International Index of Erectile Function, IPSS = International Prostate Symptom Score, LSWT = low intensity shock wave therapy, NIH-CPSI = National Institute of Health Chronic Prostatitis Symptom Index, QoL = quality of life, SHIM = Sexual Health Inventory for Men.

∗ Wilcoxon signed rank.

**Table 2:** Summary of changes in uroflowmetry following low intensity shock wave therapy.

Variables	Peak flow rate (Q_max_)	Void volume (ml)	Voiding time (s)
Pre-LSWT	21.21 ± 10.77	436.17 ± 185.35	44.41 ± 25.26
Post-LSWT	20.58 ± 7.84	472.85 ± 169.95	48.34 ± 22.36
*P* value	.336	.057	.757

LSWT = low intensity shock wave therapy.

## 4 Discussion

The current study has revealed the improvement of clinical symptoms of chronic pelvic pain syndrome (CPPS) following low intensity shock wave therapy (LSWT) administration. However, no statistically significant changes were observed for the Sexual Health Inventory for Men (SHIM) and uroflowmetry parameters.

From the literature, reports on the efficacy of LSWT to improve CPPS symptoms are generally positive. Apart from being an easy, safe, and anesthesia-free procedure to treat CPPS, these studies also reported LSWT to show significant improvements in pain and quality of life of the patients.^[20–29]^ For example, a study conducted by Li et al^[20]^ in 2020 reported the use of LSWT to treat patients with CPPS. The study was carried out on 32 patients who suffered from CPPS for more than 3 months. After the administration of LSWT without anesthesia via perineal approach for 4 weeks, the patients showed significant improvement in the Visual Analog Scale for pain measurement and NIH-CPSI. Meanwhile, Zhang et al^[28]^ conducted an experiment to compare the effects of LSWT on improvement of CPPS symptoms vs drug therapy using a combination of alpha blocker and anti-inflammatory agent. The results of the study showed that the 25 CPPS patients who received LSWT reported a significantly better improvement in NIH-CPSI compared to patients who received drug treatment.

The National Institute of Health Chronic Prostatitis Symptom Index (NIH-CPSI) was observed as the most common tool for assessing CPPS symptoms.^[20–29]^ This is probably due to the fact that it can capture 3 different domains of complaints related to CPPS, namely the pain experienced, the symptom of incomplete urination experienced and how CPPS affects their quality of life. Significant improvement of all domains of NIH-CPSI observed in the current study agrees with all the previous reports with LSWT and CPPS, which also indicated significant improvements in the NIH-CPSI scores.^[20–29]^ For instance, a study by Mykoniatis et al^[25]^ used NIH-CPSI as one of the parameters to measure improvement in CPPS-related symptoms following LSWT therapy. Their results demonstrated significant improvement in NIH-CPSI scores for all the 30 CPPS patients who underwent 6 sessions of LSWT compared to 15 patients who did not undergo the treatment. Similarly, Zimmermann et al^[23]^ also used NIH-CPSI to evaluate the effects of LSWT on QoL of CPPS patients. The results of their study also showed significant improvement in NIH-CPSI scores for all 30 patients who underwent LSWT following standardized follow-up evaluation performed at 1, 4, and 12 weeks after the treatment.

More detailed information on the CPPS related symptoms were further assessed with the International Prostate Symptom Score (IPSS). Through this instrument, severity of CPPS is depicted by the frequency, intermittency, and urgency of symptoms like incomplete voiding, straining and nocturia. In line with previous studies which reported significant improvements in IPSS following LSWT treatment,^[20–29]^ this study also shows improvement in the parameter after the treatment, indicating a significant reduction in the severity of CPPS symptoms. Furthermore, improvement in IPSS of the current study also adds to the literature on the efficacy of ESWT to significantly reduce the severity of the CPPS symptoms.^[23–25,28]^

Erectile dysfunction (ED) affects most men who are suffering from CPPS.^[2]^ Hence, the International Index of Erectile Function (IIEF) and SHIM were used to assess severity of the ED symptoms among the patients. SHIM, also known as IIEF-5, is an abridged version of the 15-items IIEF.^[30]^ It is widely used in studies investigating LSWT application on ED.^[15,30]^ The reason for using both SHIM and IIEF is that SHIM focuses on erectile function and intercourse satisfaction while the IIEF measures the impact of the erection problems on the patient's overall sex life.^[15,30]^ Besides, the SHIM questionnaire is reported to be an efficient tool to screen for ED in older patients aged between 51 and 70,^[30]^ which is relevant for this study as the mean age of the patients is around 41.9 ± 11.7 years old. In agreement with all the previous studies, LSWT significantly improves erectile function and its impact on the patient's overall sex life as represented by the IIEF score.^[21–25]^ Improvement can also be observed with SHIM score but the difference was not significant.

In the current study, parameters of uroflowmetry were included for objective assessment of the efficacy of LSWT in CPPS. The results were in accordance to the previous study by Mykoniatis et al^[25]^ whereby none of the uroflowmetry parameters were affected by LSWT treatment. This is probably due to the fact that the baseline uroflowmetry parameters were already at normal level, suggesting that the patients included had only mild urinary symptoms. Inclusion of patients with more severe urinary symptoms may result in a more significant changes following LSWT.

The exact pathophysiology of CPPS is relatively unknown. One of the manifestations of CPPS is the abnormal tone of periprostatic muscle, which may indicate abnormalities of the neuromuscular connection.^[31]^ Accordingly, the pain sensation in CPPS can be a result of endogenous generation of pain via nociceptive nerve endings and receptors. LSWT is known to modulate various cellular and molecular mechanisms by utilizing mechanotransduction system available in certain type of tissue.^[32]^ Hence, it is possible that the LSWT could hyperstimulate nociceptors within the periprostatic muscle and interrupt the process of pain generation. Further study with cellular model of the periprostatic muscle is required to confirm this hypothesis.

In the current study, LSWT was administered for 10 sessions over the course of 5 weeks. The intensity of the treatment is higher compared to previous studies where the majority employed 4 sessions of LSWT over the course of 4 weeks. From the findings, the additional sessions resulted in outcome that is comparable to previous results without causing any significant adverse effect to the patient.

However, there is a limitation in this study, which is small sample size. Only a total of 50 patients participated in the study. For the measurement of uroflowmetry outcome, data were only available for 47 patients before the LSWT treatment and 26 patients after the treatment. Therefore, study with a larger sample size should be conducted to better validate the efficacy of LSWT treatment on CPPS.

## 5 Conclusion

The LSWT administered in 10 sessions over 5 weeks presented significant improvement in terms of the patient's pain, urinary symptoms, erectile function and quality of life related to CPPS as demonstrated by the improvement in NIH-CPSI, IIEF, and IPSS outcome.

## Acknowledgments

We would like to thank all the participants who consented to complete our questionnaire.

## Author contributions

**Conceptualization:** Datesh Daneshwar, Abid Nordin.

**Data curation:** Datesh Daneshwar, Abid Nordin.

**Formal analysis:** Datesh Daneshwar, Abid Nordin.

**Project administration:** Datesh Daneshwar.

**Supervision:** Datesh Daneshwar.

**Writing – original draft:** Abid Nordin.

**Writing – review & editing:** Abid Nordin.
